# London 2012- a survey of the impact of headache on UK elite athletes

**DOI:** 10.1186/1129-2377-14-S1-P2

**Published:** 2013-02-21

**Authors:** R Hall, L Reddin, S Weatherby

**Affiliations:** 1 Peninsula Headache Clinic, UK

## Background

It is well understood that to maximise performance in sport it is important to optimise all possible variables. Headache is a very common symptom, while migraine affects between 10-15% of the population. Although athletes are frequently subject to large numbers of potential migraine triggers (exertion, stress, fatigue, dehydration), there are relatively little data examining the impact of headache conditions on elite athletes.

## Objectives

In order to investigate the prevalence and impact of headache on the performance of elite athletes we are undertaking a joint study together with the Faculty of Sports and Exercise Medicine UK.

## Methods

A questionnaire has been formulated together with the British Association for the Study of Headache (BASH), and the Faulty of Sports and Exercise Medicine. It will be submitted to various professional sports and athletic associations. To date the British Lawn Tennis Association, British Horse Racing Authority and Football Association (UK) have been approached.

## Results

At present, preliminary results are available from 30 questionnaires recently received via the British Lawn Tennis Association and British Horse Racing Authority.

57% (17/30) of respondents reported that headaches limited optimal performance in their sport (12/22 males; 5/8 females), although only one respondent fulfilled ID migraine criteria.

Of headache sufferers, 10% (2/17) stated that headaches were problematic before or during major competitions, 41% (7/17) did not have an effective method to manage their headaches and 35% (6/17) stated they would welcome more help to manage their headaches.[2]

## Conclusion

These preliminary results showed a significant proportion of elite athlete respondents suffer performance limiting headaches. While further data should quantify this more accurately, headache may be an under-addressed issue in elite athletes. Healthcare professionals working in sports medicine may find that paying greater attention to the impact and management of headache disorders would improve the performance of elite athletes under their care.
